# Glutaredoxin 1 Deficiency Leads to Microneme Protein-Mediated Growth Defects in *Neospora caninum*

**DOI:** 10.3389/fmicb.2020.536044

**Published:** 2020-08-31

**Authors:** Xingju Song, Xu Yang, Yangfei Xue, Congshan Yang, Kaijian Wu, Jing Liu, Qun Liu

**Affiliations:** ^1^National Animal Protozoa Laboratory, College of Veterinary Medicine, China Agricultural University, Beijing, China; ^2^Key Laboratory of Animal Epidemiology of the Ministry of Agriculture, College of Veterinary Medicine, China Agricultural University, Beijing, China

**Keywords:** *Neospora caninum*, glutaredoxin 1, microneme proteins, reactive oxygen species, invading, egression

## Abstract

*Neospora caninum* is an obligate intracellular protozoan parasite that infects a wide range of mammalian species and causes spontaneous abortion in cattle. *N. caninum* is exposed to oxidative stress during its life cycle. Oxidoreductase is crucial for parasite response to the environmental stresses. Glutaredoxins (Grxs) are small oxidoreductases of the thioredoxin family proteins that catalyze thiol-disulfide exchange reactions by utilizing electrons from the tripeptide glutathione (γGlu-Cys-Gly; GSH). Grxs are key elements in redox signaling and cell signal transduction. However, Grxs are an unexplored set of oxidoreductases in *N. caninum*. Here, we identified two cytoplasm located glutaredoxin domain-containing proteins (NcGrx1 and NcGrx3) in *N. caninum*. To better understand the functions of these Grx proteins, we generated NcGrx1 and NcGrx3 deficiency and overexpression strains. The deletion or overexpression of NcGrx3 had no significant effect on the growth of *N. caninum in vitro* and *in vivo*. NcGrx1 knockout parasites displayed a significant growth defect, which was due to the influence on invasion and egress abilities. Moreover, NcGrx1 deficiency decreased the ratio of reduced glutathione (GSH) to oxidized glutathione (GSSG) (GSH/GSSG ratio), caused a significant accumulation of hydroxyl radical in parasites, and an increase in apoptotic cells under oxidative stress (H_2_O_2_) condition. To determine the cause of growth defects in ΔNcGrx1, we examined the transcription levels of various invasion-egress related genes as measured by qPCR. We found a significant decrease in MIC1, MIC4, and MIC6 genes. Further investigation found that the secretion of MIC1, MIC4, and MIC6 proteins was significantly affected. Collectively, Ncgrx1 is important for microneme protein-mediated parasite growth, and maybe a potential intervention target for the *N. caninum*.

## Introduction

*Neospora caninum* (*N. caninum*) is an obligate intracellular apicomplexan parasite causing neosporosis, which results in spontaneous abortion in cattle and neural system dysfunction in dogs. Neosporosis is widely prevalent worldwide, causing huge economic losses to the dairy farming industry (Dubey, 1999; Hall et al., 2005; Lyon, 2010). Like *Toxoplasma*, the lytic cycle of *N. caninum* tachyzoites involves invasion, replication, and egress. Primarily, tachyzoites enter the host cell through an active invasion mechanism. Subsequently, the tachyzoites are surrounded by a parasitophorous vacuole (PV) membrane and replicate inside the established PV. Eventually, egress is triggered, resulting in host cell destruction (Blader et al., 2015; Frénal et al., 2017). Of the three processes, invasion and egress are particularly important due to tissue destruction in the infected host cell. Consequently, identification of the proteins required for the invasion and egress processes is important for the development of novel therapeutics against neosporosis. The secretion of motility-associated motors and adhesins from the micronemes are required for the initial invasion, egress and movement to a new host cell and its subsequent invasion (Frénal et al., 2017).

During the life cycle, *N. caninum* is exposed to oxidative stress, mainly from the aerobic metabolism products of the parasite or the host immune system (Piacenza et al., 2009). To maintain redox balance in different stages, parasites develop complex redox networks (Mohring et al., 2016). The thiol redox state is a mediator in transcription, membrane channels, metabolic enzymes, and phosphorylation signaling pathways. Glutaredoxins (Grxs) are ubiquitous small thiol-disulfide oxidoreductases that maintain redox homeostasis in cells together with the thioredoxin family. Grxs play crucial roles in redox-dependent signaling pathways by utilizing glutathione (GSH) as a direct electron donor (Lillig et al., 2008). However, knowledge of Grxs in parasites is limited.

GSH biosynthesis is important for the blood-stage survival of *Plasmodium falciparum* (*P. falciparum*) (Patzewitz et al., 2012), and GSH transport has vital functions for the chloroquine resistance of *P. falciparum* (Rahlfs and Becker, 2006; Patzewitz et al., 2013). To further elucidate redox-based parasite-host cell interactions and mechanisms of antimalarial action, the redox-sensitive green fluorescent protein was coupled to human Grx 1 (hGrx1-roGFP2). A targeted transfer of hGrx1-roGFP2 into the *P. falciparum* cytoplasm, mitochondria, or apicoplast was utilized to detect pH values and glutathione-dependent redox potentials in different subcellular compartments (Mohring et al., 2016, 2017; Jortzik and Becker, 2012). Similarly, antioxidation mechanisms in trypanosomes are critical to parasite survival in host cells after infection (Comini et al., 2013; Márquez et al., 2014). *Trypanosoma cruzi* Grx (TcrGrx) is linked to apoptosis-like cell death in *T. cruzi* infections. The overexpression of TcrGrx increases the general resistance against oxidative damage and intracellular replication of the amastigote stage (Márquez et al., 2014). *T. brucei* Grx1 (TbGrx1) plays a key role in regulation of the thermotolerance of the parasites (Musunda et al., 2015). In the bloodstream stage, TbGrx2 is not essential *in vitro* or *in vivo*, but under fever-like conditions in the mammalian host, TbGrx2 deficiency leads to an increase in thermotolerance. In the procyclical stage, TbGrx2 deficiency significantly affects the morphology of the parasite and leads to irreversible proliferation arrest (Ebersoll et al., 2018).

Grxs are essential in the redox system, however, to our knowledge, no information on *N. caninum* Grxs is available to date. Herein, the identification and characteristics of NcGrxs are described. Two cytoplasmic NcGrxs (NcGrx1 and NcGrx3) were identified by adding 3 × HA tags in the C-terminal of NcGrxs. The NcGrx3 were dispensable for growth, while the NcGrx1 was important for *N. caninum* growth *in vitro.* Furthermore, the loss of NcGrx1 caused the decrease of GSH/GSSG ratio, excessive hydroxyl radical accumulation, induction of apoptosis, and growth-inhibition of parasites under oxidative stress (H_2_O_2_) condition. NcGrx1 deficiency resulted in the transcription downregulation of MIC1, MIC4, and MIC6 genes, and a marked reduction of the secretion of micronemal proteins, which significantly affected the invasion and egress processing of the parasite.

## Materials and Methods

### Ethics Statement

The animal experiments performed in strict accordance with the recommendations of the Guide for the Care and Use of Laboratory Animals of the Ministry of Science and Technology of China. All experimental procedures were approved by the Institutional Animal Care and Use Committee of China Agricultural University (under the certificate of Beijing Laboratory Animal employee ID: 18049). The mice were humanely euthanized by cervical dislocation after anesthetization by subcutaneous injection of atropine (0.02 mg/kg) when they were unable to reach food or water for more than 24 h and lost 20% body weight. The mice that remained healthy after infection were raised to the end of their lives.

### Parasites and Cell Culture

HFFs (Human foreskin fibroblasts) were purchased from the American Type Culture Collection (Manassas, VA, United States) and cultured in Dulbecco’s modified Eagle’s medium (DMEM) supplemented with 10% fetal bovine serum (FBS). The *N. caninum* wild-type (WT) strain (Nc1) were used as parental parasites for genetically engineered strains. Parasites were grown *in vitro* by serial passage on HFF cells using DMEM supplemented with 2% FBS at 37 °C and 10% CO_2_.

### Bioinformatic Analysis of NcGrxs

The complete gene sequences of NcGrx1 (NCLIV_038390) and NcGrx3 (NCLIV_015460) were downloaded from ToxoDB^1^. The ExPASy Proteomics Server^2^ and SMART^3^ were used to predict conserved domains and motif analysis. Amino acid sequence alignment was performed using Clustal X software version 1.83. Three-dimensional structural modeling was performed using the SWISS-MODEL server^4^, and the model was based on the crystalline structure of *P. falciparum* Grx1 (PDB accession code: 4mzc.1), which has a resolution of 0.9 Å (Li et al., 2015). PyMOL 2.3^5^ was used to mark possible GSH binding sites and select pocket coordinate positions. AutoDock Vina^6^ was used for molecular docking of GSH and NcGrx1. The key amino acid residues interacting with GSH on NcGrx1 and the corresponding interaction forces were predicted by Discovery Studio v4.5 (Accelrys, San Diego, CA, United States).

### Construction of Transgenic Parasite Lines

The EuPaGDT Library in ToxoDB was used to design the gRNA targeting sites of plasmid pCRISPR-CAS9-Grx1 and pCRISPR-CAS9-Grx3. The basic plasmid template was pSAG1-Cas9-NcU6-sgRNA, which was preserved in the Key Laboratory of Animal Parasitology (Beijing City, China). The plasmid construction of pCRISPR-CAS9-Grx1 was performed as previously described (Yang C. et al., 2018). The Cas9 was amplified with Cas9-primer (Supplementary Table S1). The upstream and downstream fragments containing gRNA sequences were amplified and ligated by seamless cloning (Vazyme Biotech, Co., Ltd., Nanjing). The plasmid construction of pCRISPR-CAS9-Grx3 was consistent with the pCRISPR-CAS9-Grx1.

To construct the pTCR-NcGrx1 KO, the 3’ flanking and 5’ flanking sequences of NcGrx1 were amplified from the genomic DNA of Nc1 parasites. To disrupt the NcGrx1 locus, the chloramphenicol resistance gene (CmR) and red fluorescence protein gene (RFP) were designed to insert the 3’ flank and 5’ flank of NcGrx1 regions and ligated into the plasmid backbone pTCR-CD. The pTCR-NcGrx1 KO and pCRISPR-CAS9-Grx1 plasmids were co-transfected into Nc1 parasites and screened by chloramphenicol. The monoclonal screening was carried out by a limited dilution method with reference to previous studies (Yang C. et al., 2018). The monoclonal parasites were identified by PCR followed by sequencing. The construction of pTCR-NcGrx3 KO parasites were consistent with the above.

To obtain the NcGrx1-HA parasites, we constructed pLIC-HA-DHFR-NcGrx1 plasma for inserting a 3 × HA tag into the NcGrx1 gene 3’ end. The 3’ flank and 5’ flank regions of NcGrx1 were amplified from the genomic DNA of the Nc1 parasites and directly ligated into the plasmid backbone pLIC-HA vector. The construction strategy of pCRISPR-CAS9-Grx1-HA was consistent with the above description of pCRISPR-CAS9-Grx1. The gRNA was obtained from 3’ regions of NcGrx1. The pLIC-HA-DHFR-NcGrx1 and pCRISPR-CAS9-Grx1-HA plasmids were co-transfected into Nc1 parasites and screened by pyrimethamine.

To complement NcGrx1-deficient parasites, the CRISPR/CAS9-UPRT specific gRNA was used for the targeted disruption of the UPRT gene, which was replaced with the cDNA sequence of NcGrx1 (Yang C. et al., 2018). The UPRT locus-targeted homologous recombinant plasmid, including p5’UPRT-Tubulin promoter-DHFR-Grx1-HA-3’UPRT and the CRISPR/CAS9-UPRT plasmid, were co-transfected into the NcGrx1 knockout strain (ΔNcGrx1). Fluorodeoxyribose (FUDR) and pyrimethamine were used for positive strain screening. The construction strategy for the over-expression strain was the same as the complementary strain. The homologous recombinant plasmid and CRISPR/CAS9-UPRT plasmids were co-transfected into Nc1.

Finally, we achieved the knockout strains (ΔNcGrx1 and ΔNcGrx3), over-expression strains (NcGrx1 OE and NcGrx3 OE), endogenous marker strains (NcGrx1 HA and NcGrx3 HA), and NcGrx1 complementary strain (iΔNcGrx1).

### Western Blot

The western blots were performed as previously reported (Yang C. et al., 2018). Freshly isolated parasites were collected and purified by filtration through 5 μm membrane filtration and lysed with RIPA buffer (Huaxinbio, Beijing). The primary antibodies used were mouse anti-HA (MAb, 1:5000, Sigma), anti-Actin (1:6,000), anti-MIC1 (1:500), anti-MIC4 (1:400), anti-MIC6 (1:500), anti-MIC2 (1:500), anti-MIC3 (1:1000), and anti-MIC8 (1:500).

### Immunofluorescence Assay

Immunofluorescence assays (IFA) for subcellular localization were carried out as previously described (Yang C. et al., 2018). Briefly, tachyzoites infected HFFs were fixed by 4% paraformaldehyde (PFA) followed by treatment in 0.25% Triton X-100. Samples were incubated with primary mouse anti-HA (1:50), mouse anti-MIC1 (1:200), mouse anti-MIC4 (1:100), mouse anti-MIC6 (1:200), or rabbit anti-SRS2 (1:400) for 1 h. Then, secondary FITC- or Cy3-conjugated antibodies were used for labeling. DNA was stained with Hoechst 33258 (Sigma, United States). The images were obtained using a Leica confocal microscope system (Leica, TCS SP52, Germany).

### Plaque Assay

HFFs growing in 12-well plates were infected with 300 freshly harvested tachyzoites and incubated 9 days undisturbed. Subsequently, infected HFFs were fixed by 4% PFA and stained with 0.2% crystal violet solution. The plaque area was counted by pixel using Photoshop C6S software (Adobe, United States), and the data were compiled from three independent experiments.

### Invasion Assay and Intracellular Replication Assay

About 1 × 10^5^ parasites were inoculated on HFF cells in 12-well plates. After 1 h, the uninvaded parasites were removed and continuously cultured for 24 h. Then, parasites were fixed with PFA and stained by IFA using rabbit anti-SRS2 antibodies and Hoechst. For the proliferation assay, the numbers of parasites per vacuole for each strain were determined by counting at least 100 vacuoles using a fluorescence microscope (Olympus Co., Japan). Three independent experiments were performed. For the invasion assay, the percent of invasion was represented as numbers of vacuoles per host cell. Three independent experiments were performed.

### Egress Assay

Parasites were inoculated onto 12-well plates for 36 h. The egress was triggered with 2 μM of Ca^2+^ ionophore A23187 (Sigma, United States) for 3 min at 37°C before fixation with PFA (Williams et al., 2015). The IFA was performed using mouse anti-NcSRS2 antibodies. The ratio of 100 randomly selected ruptured vacuoles/whole vacuoles was counted per slide. Three independent experiments were performed.

### *N. caninum* Mouse Infection

BALB/c mice purchased from Merial Animal Health Co., Ltd. (Beijing, China) and raised in under a barrier environment in sterile cages and fed with sterilized food and clean water *ad libitum*. Animals were acclimated to these conditions for 1 week prior to the experiment. BALB/c mice (five mice per strain) were infected intraperitoneally with 8 × 10^6^ parasites. The period for observing the survival was 30 days.

### Cultivation of *N. caninum* Under Oxidative Stress

ΔNcGrx1, NcGrx1 OE, and WT parasites were grown with 50 μM H_2_O_2_, 100 μM H_2_O_2_, and 200 μM H_2_O_2_, respectively, to evaluate the function of NcGrx1 under oxidative stress. Differences in the proliferation of parasites were observed using IFA.

### TUNEL Assay

TUNEL assays were performed using an apoptosis detection kit according to the manufacturer (Vazyme Biotech, Co., Ltd., Nanjing). Briefly, ΔNcGrx1, NcGrx1 OE, and WT parasites were grown with 100 μM H_2_O_2_ in HFF cells. After 24 h, the parasites were purified and fixed on coverslips. Then parasites were incubated in the TUNEL reaction mix with the terminal deoxynucleotidyl transferase (TdT) enzyme. Anti-SRS2 polyclonal antibodies were used to stain the shape following the IFA protocol, as previously mentioned. A total of 100 parasites were counted to determine the number of TUNEL-positive parasites.

### Detection of Hydroxyl Radical

The parasites were grown under normal condition or 100 μM H_2_O_2_ condition in HFF cells. After 24 h treatment, the parasites were purified and washed. The concentration of hydroxyl radicals was detected by hydroxyl radical detection Kit using hydroxyphenyl fluorescein (2-[6-(4′-Hydroxy) phenoxy-3H-xanthen-3-on-9-yl] benzoic acid, HPF) according to the manufacturer’s instructions (GENMED SCIENTIFICS INC, United States). Finally, 10,000 parasites were analyzed by a flow cytometry.

### GSH and GSSG Determination

Parasites were grown under normal condition or 100 μM H_2_O_2_ condition. After 24 h treatment, 1 × 10^7^ parasites of each strain were harvested and washed twice with PBS. Then parasites were lysed by frozen in liquid nitrogen and thawed at 37°C for three circles. The supernatant of each sample was collected for GSH and GSSG measurement by GSH and GSSG Assay Kit according to the manufacturer’s instructions (Beyotime, China).

### HED Assay

NcGrx1 activity was evaluated using β-hydroxyethyl disulfide (HED) assay (Holmgren and Aslund, 1995). The activity was assayed as the decrease in absorption at 340 nm at 25°C. The reaction mixture consists of 100 mM potassium phosphate, 1 mM EDTA, pH 7.0, 200 μM NADPH, 1 mM GSH, 5 μg glutathione reductase (Sigma Aldrich, St. Lois, MO, United States), and 1 mM HED in a final volume of 1 mL. After incubation at 25°C for 3 min, mixed disulphide between HED and GSH was formed. Then the reaction was started by additional of 50–1200 nM recombinant NcGrx1 (rNcGrx1). The NADPH consumption was detected at 340 nm. The kinetic properties of rNcGrx1 (600 nM) were determined using 0.05–2 mM HED. One unit of activity is defined as the consumption of 1 μM of NADPH per minute. Kinetic parameters (V_max_, K_m_ and K_cat_) were calculated using GraphPad Prism^®^ software (San Diego, CA, United States).

### RNA Sequencing and Differential Gene Expression Analysis

Total RNA from freshly egressed Nc1 and ΔNcGrx1 parasites was extracted using TRIZOL (Sigma). The *N. caninum* F-actin subunit beta gene (ToxoDB: NcLIV_061190, Nc-Actin) was selected as the endogenous reference gene. The transcriptional levels of MIC1, MIC2, M2AP, MIC3, MIC4, MIC6, MIC8, AMA1, SUB1, RON2, NTPase3, and CDPK genes in Nc1 and ΔNcGrx1 parasites were detected using Real-time quantitative PCR. Real-time quantitative PCR was performed using AceQ qPCR SYBR Green Master Mix (Vazyme Biotech, Co., Ltd., Nanjing). Data were determined by Roche LightCycler 480 (Roche, Basel, Switzerland) and normalized to Nc-Actin expression levels.

### Microneme Proteins Secretion

Microneme proteins secretion assays were performed according to previously described procedures (Rodríguez-Manzaneque et al., 2002). Briefly, tachyzoites were purified and washed with DMEM. After resuspension in 200 μL DMEM, parasites were treated with 2 μM A23187 or 10 mM dithiothreitol (DTT, Calbiochem) for 20 min at 37°C. Excreted/secreted antigen (ESA) fractions were collected by centrifugation 15 min at 1000 *g*. The ESA and precipitation fractions were subjected to western blotting to assess microneme protein secretion, respectively. The secretion of proteins was quantitatively evaluated by ImageJ.

### Statistical Analysis

Graphs were created and statistical analyses were conducted using Graph Pad Prism (San Diego, CA, United States). Graphs represent means, and error bars represent standard errors of means. All data were analyzed with One-way ANOVA and the two-tailed Student’s *t*-test. *P*-values are represented by asterisks in figures as follows: ^∗^*p* < 0.05, ^∗∗^*p* < 0.01, and ^∗∗∗^*p* < 0.001. We consider all *p* < 0.05 to be significant.

## Results

### Sequence Characterization and Phylogenetic Analysis of NcGrxs

**FIGURE 1:**
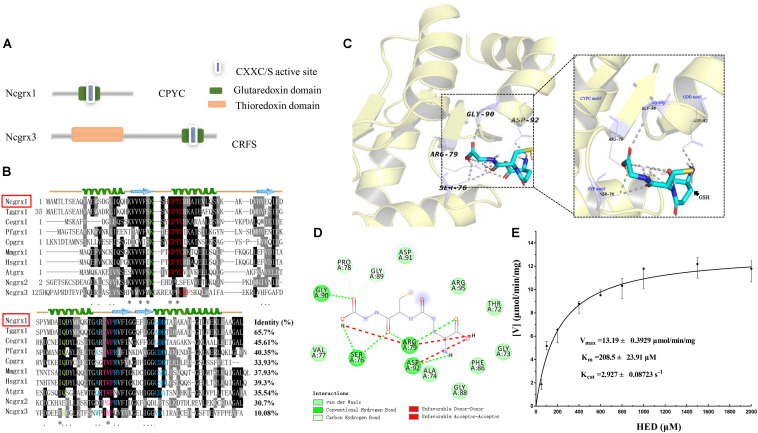
Sequence-structural alignment of glutaredoxin proteins of *Neospora caninum.* **(A)** The model pattern of the two glutaredoxin family proteins containing the glutaredoxin domain in *Neospora caninum*. **(B)** Alignment of the deduced amino acid sequence of NcGrx1 with homologs from other species. The percent homology of NcGrx1 with each glutaredoxin is shown at the end of the alignment. Regions of high identity and similarity between glutaredoxin sequences are shown as black and gray columns, respectively. The “*” represent highly conserved amino acid residues. The active-site residues CXXC/CXXS are marked with a red letter. The residues involved in interactions with Gly, Cys, and ^γ^Glu of GSH are highlighted in green, pink and red, and blue, respectively. The predicted secondary structure of NcGrx1 is displayed above the alignment. **(C)** The 3D structure of NcGrx1 shown as the cartoon was modeled based on the crystalline structure of *Plasmodium falciparum* Grx1 (PDB accession code: 4mzc.1). The GSH-binding motifs (Lys and Gln, CPYC, SVP, Gly-Gly, and GDD) are displayed on the 3D structures. PyMol was used to mark possible GSH binding sites and select pocket coordinate positions. The key amino acid residues interacting with GSH on NcGrx1 were predicted by AutoDock Vina and highlighted in purple (SER76, ARG-79, GLY90, and ASP-92). **(D)** The corresponding interaction forces of amino acid residues interacting with GSH on NcGrx1 were predicted by Discovery Studio 4.5. **(E)** HED assay. The activity of recombinant NcGrx1was assayed as the decrease in absorption at 340 nm at 25°C. One unit of activity is defined as the consumption of 1μM of NADPH per minute. Kinetic parameters (V_max_, K_m_, and K_cat_) were calculated using GraphPad Prism^®^ software. The data are the mean ± SD of three independent experiments.

To obtain information on glutaredoxins in *N. caninum*, we used the ToxoDB genomic resource database to search for possible glutaredoxin-related genes. Five glutaredoxin-containing genes were found in *N. caninum*, and two of these genes (NCLIV_038390 and NCLIV_015460) were predicted to be located in the cytoplasm. NCLIV_038390 showed the greatest similarity (47%) with glutaredoxin 1 from *Homo sapiens* (HsGrx1), hereafter named NcGrx1. NCLIV_015460 was the most similar to mammalian glutaredoxin 3, hereafter named NcGrx3. The basic information on NcGrx1 and NcGrx3 were obtained from ToxoDB (Supplementary Table S1). Sequence analysis showed that NcGrx1 had a Cys-Pro-Tyr-Cys (CPYC) active site on the glutaredoxin domain, which is a classic dithiol motif. NcGrx3 contained a thioredoxin domain and a CRFS active site on the glutaredoxin domain (Figure 1A), which is classified as a monothiol Grx.

Sequence alignment revealed that NcGrx1 contains GSH-binding motifs (CPYC, SVP, GDD motifs, and Lys and Gln/Arg residues) (Figure 1B), which exposed a larger hydrophobic binding pocket (Figure 1B). Notably, the TVP and CSD motifs on NcGrx1 are mutated to SVP and GDD as compared with the Grx1 from host cells (*Homo sapiens* and *Mus musculus*). The three-dimensional structure of NcGrx1 was modeled using the structural template of *P. falciparum* Grx1 (PfGrx1) using the SWISS-MODEL server. NcGrx1 was conserved, as judged by structural modeling, and included a β-sheet of four strands surrounded by three α-helices (Figures 1B,C). The Ser76 in the SVP motif, Arg79, Gly90 in Gly-Gly, and Asp92 in the GDD motif were predicted to directly interact with GSH according to conventional hydrogen bonding (Figure 1D). The oxidoreductase activity of rNcGrx1 was assessed by the HED assay. The NADPH reduction was directly correlated with rNcGrx1concentrations, which showed rNcGrx1 have a Grx-specific activity for HED (Supplementary Figure S2). The V_max_, K_m_, and K_cat_ of rNcGrx1 were 13.19 ± 0.3929 μmol/min/mg, 208.5 ± 23.91 μM and 2.927 ± 0.08723 s^–1^, respectively (Figure 1E). Therefore, the K_cat_/K_m_ can be calculated as 1.4038 × 10^4^ M^–1^s^–1^.

### NcGrxs Localizes to the Cytosol

**FIGURE 2:**
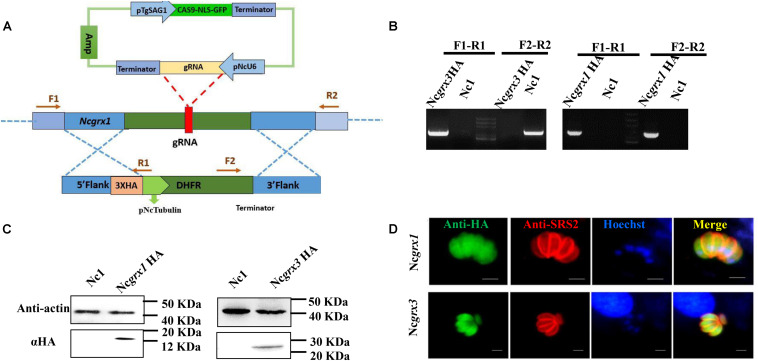
The cellular localization of NcGrxs. **(A)** Strategy for construction of the NcGrx1-HA and NcGrx3-HA parasites. **(B)** The insertion of 3 × HA-tagged NcGrxs in parasites was confirmed by PCR. **(C)** Western blot confirmed the expression of HA-tagged NcGrxs in parasites. Actin was used as a control. **(D)** Immunofluorescence assays analysis of NcGrxs localization. αHA was used to detect the NcGrxs-HA (green), and SRS2 (red) served as a parasite surface marker (bar = 5 μm).

To reveal the localization of NcGrxs proteins, we constructed HA epitope-tagged NcGrx1 and NcGrx3 in the *N. caninum* wild-type (WT) strain (Nc1) parasites (Figure 2A). The polymerase chain reaction (PCR) confirmed the insertion of endogenous tags correctly (Figure 2B). Western blot analysis using an anti-HA antibody showed a single band of the expected size for each protein (Figure 2C). Immunofluorescence assays (IFAs) showed that NcGrx1 and NcGrx3 were distributed throughout the cytosol (Figure 2D).

### NcGrx1 Is Important for the Growth of *N. caninum*

**FIGURE 3:**
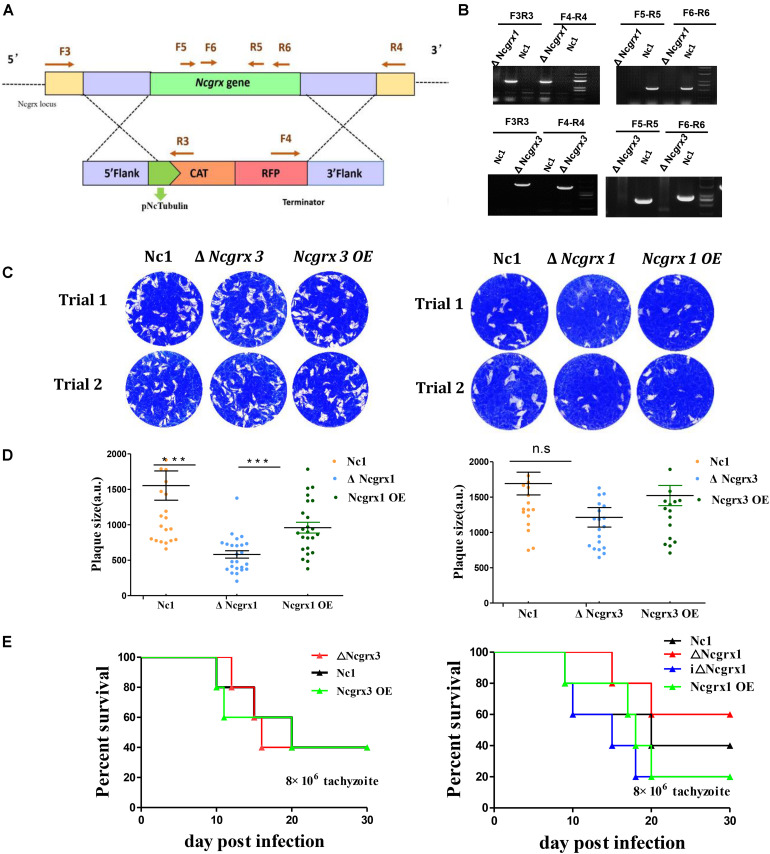
Lack of NcGrx1 adversely affects the growth of parasites. **(A)** Strategy for construction of ΔNcGrx1 and ΔNcGrx3 strains. **(B)** PCR identification of ΔNcGrx1 and ΔNcGrx3 parasites using the primers indicated above. **(C)** Plaque assays comparing the growth of wild-type, knockout, and overexpression parasites. Each well was infected with 300 parasites, and plaques were stained for 9 days. The plaque areas were counted by randomly selecting at least 20 plaques and measured using the pixel point in Photoshop C6S software (Adobe, United States). The data were compiled from three independent experiments. **(D)** Quantification of plaque area sizes. Analysis of the plaque area was performed using *t*-test. **(E)** Mouse survival after infection with different strains. BALB/c mouse (*n* = 5) were injected intraperitoneally with 8 × 10^6^ doses of parasites. Statistical analysis was performed using survival curve of Graph Pad Prism (SAS Institute Inc., United States). ***p < 0.001.

NcGrx1 and NcGrx3were localized in the cytoplasm; therefore, we sought to determine whether the NcGrxs are necessary for parasite growth. To independently assess the functions of NcGrx1 and NcGrx3, we generated the ΔNcGrx1, ΔNcGrx3, and iΔNcGrx1 parasites, respectively (Figure 3A and Supplementary Figure S1A). All strains were validated using PCR (Figure 3B and Supplementary Figure S1B). The plaque assay is used to comprehensively evaluate the growth of *N. caninum* in the entire lytic cycles. The plaque area can reflect the growth ability of the parasites. Thus, we detected the effects of NcGrx1 or NcGrx3 deficiencies on parasite growth *in vitro* by monitoring the formation of plaques. A significant reduction (*p* < 0.001) in plaque formation size was observed in ΔNcGrx1 parasites as compared with Nc1 parasites (Figures 3C,D), and the plaque formation was restored in the iΔNcGrx1 parasites (Supplementary Figure S1C). Dissimilarly, the deletion or overexpression of NcGrx3 had no significant effect on the growth of *N. caninum in vitro* (Figures 3C,D). To further explore the effect of NcGrx1 or NcGrx3 deficiency on parasite growth *in vivo*, the parasites were injected in BLAC/c mice. The mortality rate of ΔNcGrx1 infected mice was reduced by 20%, and the survival time is prolonged 6 days (Figure 3E). No statistically significant differences in survival were seen, as mice infected with Nc1, ΔNcGrx3 or NcGrx3 OE strains (Figure 3E).

### Loss of NcGrx1 Affects the Invasion and Egress of Parasites

**FIGURE 4:**
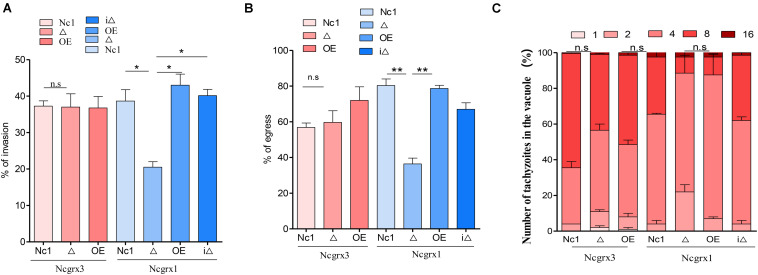
Loss of NcGrx1 perturbs the invasion and egress of parasites. **(A)** 1 × 10^5^ parasites were inoculated on HFF cells in 12-well plates and cultured for 24 h. The IFA was performed with anti-NcSRS2 antibodies and Hoechst. Invasion ratio of wild-type, knockout, overexpression, and complementary parasites was grounded on the number of parasite-infecting cells divided by the number of total cells in one horizon. Asterisks indicated statistically significant results (*p* < 0.05 as determined by *t*-test). Data are the mean ± SD (error bars) of three independent experiments. **(B)** The egress ability of parasites was assessed after treatment with calcium ionophore A23187. IFA was used to detect the integrity of parasitophorous vacuole (PV). The average number of ruptured PV was determined by counting random 100 vacuoles per slide. **(C)** Intracellular replication of different parasite strains was compiled from three separate assays, and in each assay 100 total PVs of each strain were counted. Statistical analysis showed no change. Data were identified by Chi-square analysis. Asterisks indicated statistically significant results. **p < 0.01 and *p < 0.05.

The growth of *N. caninum* tachyzoites in cells involved a complete set of the lytic cycles, including invasion, intracellular replication, and egress (Blader et al., 2015). Reduction in plaque formation can be caused by impairment of one or more steps of the lytic cycle. Thus, we next sought to investigate the role of NcGrx1 in the lytic cycle biology of *N. caninum*. We primarily assessed parasite invasion processes, which showed a significant weakening (∼40%, *p* < 0.5) of the host cell invasion in ΔNcGrx1 parasites as compared to Nc1 (Figure 4A). Then, the calcium ionophore A23187 was used to assess the egress ability of the parasites. The results showed that the deletion of NcGrx1 altered the egress ability from the host cell after 3 min of stimulation (Figure 4B). However, it does not affect the final egress of the parasites. When the stimulus exceeded 5 min, all parasites were egressed (data not shown). No significant difference in the intracellular replication rates was seen between the parental and knockout strains (Figure 4C). These results showed that the plaque formation size reduced in ΔNcGrx1 parasites is specifically due to impairment of the invasion and egress process.

### NcGrx1 Deficiency Impairs Microneme Protein Secretion

**FIGURE 5:**
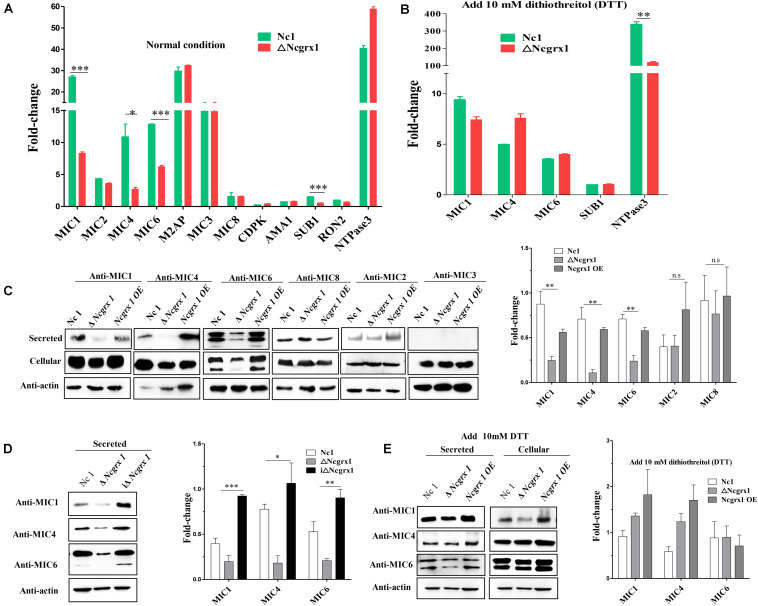
NcGrx1 deficiency impairs the secretion of microneme proteins. **(A)** Quantification of the transcription levels of various invasion-egress related genes (MIC1, MIC2, M2AP, MIC4, MIC3, MIC6, MIC8, AMA1, CDPK1, SUB1, NTPase1, NTPase2, NTPase3, and RON2) transcript levels in ΔNcGrx1 and Nc1 strains by qRT-PCR under normal condition **(B)** and 10 mM DTT condition. The *N. caninum* F-actin subunit beta gene (ToxoDB: NcLIV_061190, Nc-Actin) was the endogenous reference gene. Asterisks indicated statistically significant results (***p < 0.001, **p < 0.01, and *p < 0.05 as determined by *t*-test). **(C,D)** Freshly egressed parasites were collected and treated with 2 μM A23187 for 20 min at 37°C to stimulate microneme secretion. Parasite lysate (cellular) and ESA (secreted) fractions from Nc1, ΔNcGrx1, NcGrx1 OE, and iΔNcGrx1 strains were collected and used for western blotting analysis. Nc-Actin was immunoblotted as the loading control for each strain. The secretion of proteins was quantitatively evaluated by ImageJ according to three independent experiments. Statistical analysis was performed using One-way ANOVA. **(E)** Freshly egressed parasites were collected and treated with 10 mM DTT for 20 min at 37°C to stimulate microneme secretion. Parasite lysate (cellular) and ESA (secreted) fractions from Nc1, ΔNcGrx1, and NcGrx1 OE were collected and used for western blotting analysis. The secretion of proteins was quantitatively evaluated by ImageJ according to three independent experiments.

Microneme proteins are critical for the invasion and egress process of *N. caninum* (Carruthers et al., 2000; Brecht et al., 2001; Rabenau et al., 2001; Reiss et al., 2001; Kafsack et al., 2009; Zheng et al., 2009; Li et al., 2015; Wang and Yin, 2015; Frénal et al., 2017). The loss of NcGrx1 affects the invasion and egress ability of parasites. Therefore, we next determined whether these impairments were related to microneme proteins. We first analyzed the transcriptional levels of various invasion-egress related genes, including MIC1, MIC2, M2AP, MIC3, MIC4, MIC6, MIC8, AMA1, SUB1, RON2, and CDPK. The result showed significant decreases in the transcriptional levels of the microneme proteins, including MIC1 (3.2-fold change), MIC4 (3.99-fold change), and MIC6 (2.08-fold change), and a subtilisin protease (SUB1) (2.69-fold change) in the ΔNcGrx1 compared to Nc1 parasites (Figure 5A). Interestingly, after treating the parasite with dithiothreitol (DTT), the transcriptional levels of MIC1, MIC4, MIC6, and SUB1 in ΔNcGrx1 parasites were recovered (Figure 5B). Then, we evaluated the nucleotide triphosphate–degrading enzymes 3 (NTPase3) gene, and no significant differences in the transcription levels between ΔNcGrx1 and Nc1 strains in the untreated DTT group were observed. Conversely, NTPase3 level in the Nc1 parasite was much higher (2.86-fold change) than in ΔNcGrx1 in the DTT treatment group (Figure 5B).

**FIGURE 6:**
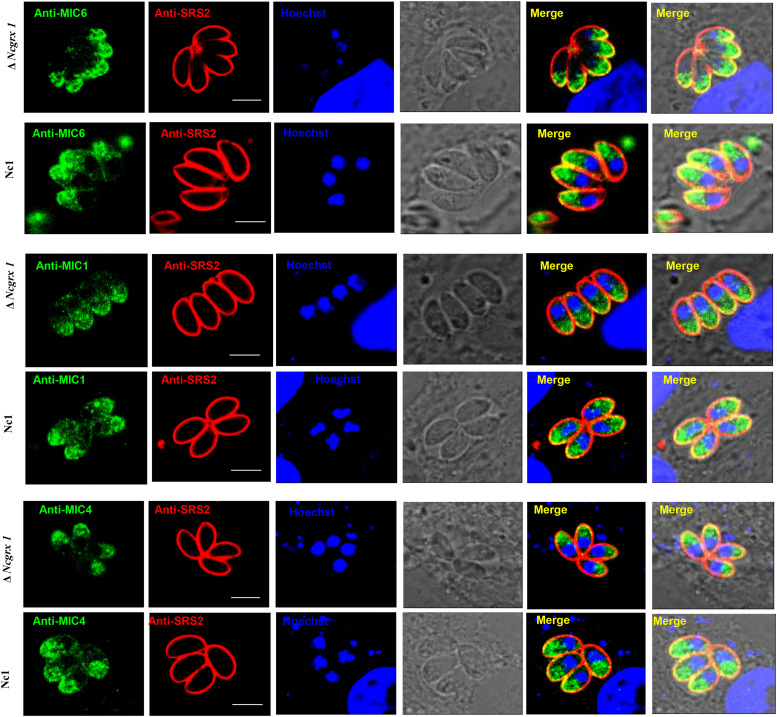
NcGrx1 deletion does not affect the location of microneme proteins. The location of MIC1, MIC4, and MIC6 proteins (green) in ΔNcGrx1 and Nc1 strains was detected by IFA. The shapes of parasites were visualized with anti-NcSRS2 (red), and the nuclear DNA was stained with Hoechst (blue). Scale bar = 5 μm.

To further study whether NcGrx1 deficiency impairs the expression and secretion of microneme proteins, we performed microneme proteins (MIC1, MIC4, MIC6, MIC2, MIC3, and MIC8) secretion assays with Ca^2+^ ionophore A23187 or DTT. The results revealed that the secretion of MIC1, MIC4, and MIC6 were significantly reduced in the ΔNcGrx1 as compared to Nc1 parasites (Figure 5C), and the secretions of these proteins were recovered in iΔNcGrx1 parasites (Figure 5D). No significant difference was observed in MIC2, MIC3, and MIC8 in the ΔNcGrx1 as compared to Nc1 parasites (Figure 5D). When stimulated with DTT, the secretion of MIC1, MIC4, and MIC6 were not affected (Figure 5E). Furthermore, we evaluated whether NcGrx1 deficiency affected the secretion of micronemes directly or indirectly through incorrect transport. Our results showed that the location of MIC1, MIC4, and MIC6 were not affected by NcGrx1 deletion (Figure 6). These results indicated that the growth defects in ΔNcGrx1 parasites *in vitro* are due to some microneme proteins (MIC1, MIC4, and MIC6) secretion deficiency.

### NcGrx1 Deficiency Caused Growth-Inhibition of Parasites Under Oxidative Stress

**FIGURE 7:**
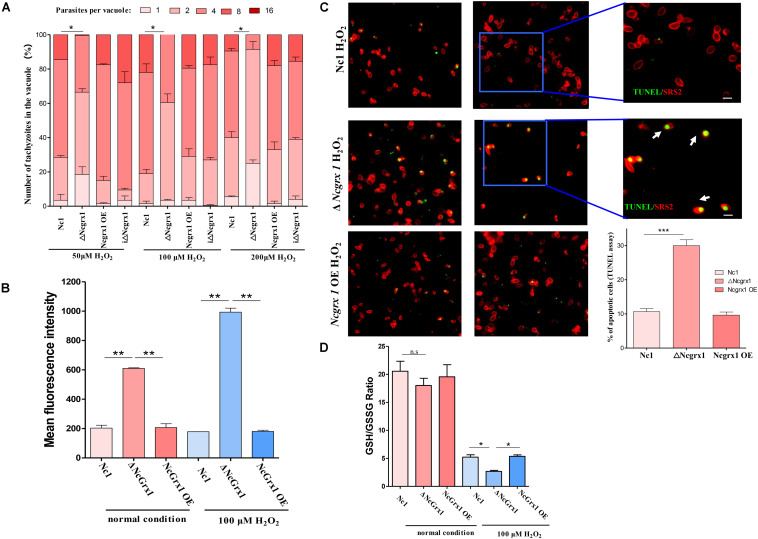
NcGrx1 deficiency caused growth-inhibition, excessive ROS accumulation and apoptosis induction of parasites under oxidative stress. **(A)** ΔNcGrx1, NcGrx1 OE, and WT parasites were grown under oxidative stress (H_2_O_2_) in HFF. The proliferation of all strains was observed by IFA. Anti-SRS2 was used as a parasite surface marker. In each assay, 100 total PVs of each strain were counted. **(B)** Hydroxyl radical levels of parasites under normal condition or 100 μM H_2_O_2_ condition were determined by FACS analysis. The mean fluorescence intensity can reflect the hydroxyl radical level in parasites. **(C)** Apoptosis of ΔNcGrx1, NcGrx1 OE, and Nc1 parasites was assessed by the TUNEL assay after treatment with H_2_O_2_. The TUNEL-positive and TUNEL-negative parasites are shown in the left panel. The ratio of apoptotic parasites was counted using Graph Pad Prism (right panel). Asterisks indicate statistically significant results as determined by the *t*-test (*p* < 0.001). Scale bar = 5 μm. Asterisks indicated statistically significant results (***p < 0.001, **p < 0.01, and *p < 0.05 as determined by *t*-test). **(D)** The parasites were grown under normal condition or 100 μM H_2_O_2_ condition1. 1 × 10^7^ ΔNcGrx1, NcGrx1 OE, and Nc1 parasites were harvested. The concentration of GSH and GSSG was quantified by GSH and GSSG Assay Kit. The GSH/GSSG ratio was calculated and represented by a bar charts according to three independent experiments. Asterisks indicate statistically significant results as determined by the *t*-test.

The effects of NcGrx1 deficiency and overexpression on the growth of parasites were observed under oxidative stress. The deletion of NcGrx1 increased the sensitivity of the parasites to oxidative stress as compared with the WT strain (Figure 7A), which indicated that NcGrx1 deficiency increased susceptibility under oxidative stress.

### NcGrx1 Deficiency Caused Hydroxyl Radical Accumulation and Induced Apoptosis in Parasites Under Oxidative Stress

Grx1 plays an important role in the ROS antioxidant system. Therefore, to examine the effects of NcGrx1 deficiency on redox homeostasis, we measured hydroxyl radical levels under normal condition or 100 μM H_2_O_2_ condition. NcGrx1 knockdown increased hydroxyl radical accumulation threefold as compared with the WT strain, and increased hydroxyl radical accumulation 5.58-fold as compared with the WT strain under oxidative stress (Figure 7B). To determine whether NcGrx1 deficiency induced apoptosis, we used the TUNEL assay to quantify the apoptotic ratios of ΔNcGrx1, NcGrx1 OE, and Nc1 strains. Approximately 30% of the ΔNcGrx1 parasites were TUNEL positive after H_2_O_2_ treatment (∼10% for Nc1 parasites) (Figure 7C), which indicated a significantly (*p* < 0.001) increased apoptosis rate. These data indicated that NcGrx1 deficiency increased the ROS level and induced apoptosis under oxidative stress. GSH is important endogenous antioxidant, which can maintain intracellular redox homeostasis by scavenging excess hydroxyl radical. Therefore, we further measured the GSH/GSSG ratio in ΔNcGrx1, NcGrx1 OE, and Nc1 parasites under oxidative stress (H_2_O_2_). The results showed that NcGrx1 deficiency decreased the GSH/GSSG ratio in tachyzoites, and no significant difference was observed under normal condition (Figure 7D).

## Discussion

Glutaredoxins are ubiquitous oxidoreductases with deglutathionylation activity. Grxs maintain cellular redox equilibrium and catalyze thiol-disulfide exchange reactions by utilizing electrons from tripeptide glutathione (^γ^Glu-Cys-Gly; GSH) (Allen and Mieyal, 2012). Grxs were classified as monothiol (CXXS) Grxs and dithiol (CXXC) Grxs depending on the number of cysteine residues present in the redox active site (Yogavel et al., 2013; Begas et al., 2017). They are involved in various physiological processes, such as transport of Fe-S clusters, apoptosis, DNA synthesis, cell proliferation, cell signal transduction, and immune defense (Lillig et al., 2008; Allen and Mieyal, 2012). However, only a few Grxs from parasites have been reported, mainly on trypanosomes and *Plasmodium* (Mohring et al., 2017; Ebersoll et al., 2018). This study characterized two *N. caninum* Grxs and explained the role of Grx1 in oxidative stress and parasite growth.

The GSH-binding motifs (CPYC, SVP, GDD motifs, and Lys and Gln/Arg residues) in NcGrx1 could form a larger hydrophobic binding pocket, which is related to the oxidized/reduced function of glutaredoxin (Yogavel et al., 2013). A comparison of the GSH-binding motifs was performed between the Grx1 sequences of mammalian hosts and *N. caninum* and showed that the TVP/CSD motifs on NcGrx1 are mutated to SVP/GDD. Interestingly, the mutated sites (Ser76 in the SVP motif and Asp92 in the GDD motif) were predicted to interact closely with GSH according to conventional hydrogen bonding. These mutant sites might provide an important foundation for the design of inhibitors with NcGrx1 as a drug target for the treatment of *N. caninum*.

Compared with other previously reported Grx1, the specific activity of rNcGrx1 for HED was higher (13.19 ± 0.3929 μmol/min/mg) than that of TbGrx1 (4.7 ± 0.1μmol/min/mg), and its affinity (K_m_ = 208.5 ± 23.91 μM) was also higher than TbGrx1 (K_m_ = 53 ± 5 μM). So, the K_cat_/K_m_ value of rNcGrx1 (1.4038 × 10^4^ M^–1^s^–1^) is very close to that of TbGrx1 (1.6 × 10^4^ M^–1^s^–1^) (Musunda et al., 2015). Moreover, the K_cat_/K_m_ of rNcGrx1 for HED is also close to *Saccharomyces cerevisiae* Grx1 (ScGrx1) (1.083 × 10^4^ M^–1^s^–1^), while a little bit higher than that of *Taenia solium* Grx1 (7.8 × 10^3^ M^–1^s^–1^) (Discola et al., 2009; Nava et al., 2019). These results indicated that rNcGrx1 have moderate oxidoreductase activity and the catalytic efficiency of rNcGrx1 for HED is similar to ScGrx1 and TbGrx1.

Deletion of NcGrx1 or NcGrx3 alone did not affect the proliferation of *N. caninum*, which is consistent with previous reports in *T. brucei*. Disruption of either TbGrx1 or TbGrx2 alone did not alter the proliferation of *T. brucei* under normal culture conditions (Ceylan et al., 2010; Musunda et al., 2015). It is noteworthy that the loss of NcGrx1 affects the growth of the parasite *in vitro* by monitoring the formation of plaques, which is caused by altering the invasion and egress abilities. Previous studies have also shown that the mutation of *Sinorhizobium meliloti* Grx1 and Grx2 lead to growth defects. The main reason for the slow growth in mutations of Grx2 may be the influence of Fe-S cluster metabolism in the nitrogenase complex assembly (Benyamina et al., 2013). In many organisms, including yeast, *Synechocystis*, *Arabidopsis thaliana*, mouse, and human, disruption of Grx caused highly sensitive to stress-induced oxidative damage, indicating that Grx plays an important role in stress adaptation (Rodríguez-Manzaneque et al., 2002; Lillig et al., 2004; Chung et al., 2005; Cheng, 2008; Mühlenhoff et al., 2010; Wu et al., 2011; Sánchez-Riego et al., 2013). In this study, the loss of NcGrx1 caused growth-inhibition of parasites under oxidative stress (H_2_O_2_) condition, which indicated that NcGrx1 is crucial for maintaining redox balance in *N. caninum* under oxidative stress. Moreover, Grx1 is important for the ROS-defense system; GSH is the main non-protein thiol anti-oxidant, which is required to scavenge ROS in cells (Brandes et al., 2014). Grx1 silencing in human cells led to a significant decline in the cellular GSH/GSSG ratio, which caused excessive accumulation of ROS (Yang F. et al., 2018). In our study, NcGrx1 knockdown increased hydroxyl radical accumulation 3-fold compared with the WT strain, while increased hydroxyl radical accumulation 5.58-fold under oxidative stress. Notably, the hydroxyl radical in the WT parasites were consistent with normal conditions, and the amount of hydroxyl radicals in ΔNcGrx1 parasites was significantly higher than that of normal condition, which suggested that the deletion of NcGrx1 results in more hydroxyl radicals that cannot be removed.

Previous research showed that Grx1 involves in apoptosis signal-regulating kinase 1 (ASK1) – mediated apoptotic signaling pathway (Ichijo et al., 1997; Kalinina et al., 2014). The reduced Grx1 binds to the ASK1, resulting in the inactivation of ASK1. ASK1 can be activated by ROS, especially by H_2_O_2_, due to the breakdown of the complex with Grx1 (Song et al., 2002; Kalinina et al., 2014). Hydroxyl radical is the most reactive oxygen species involved in many biological processes. In this study, NcGrx1 deficiency led to a significant accumulation of hydroxyl radical, and an increase in apoptotic cells under oxidative stress (H_2_O_2_) condition. Thus, it is possible that the ASK1 is activated by excessive ROS under oxidative stress, and no more new reduced Grx1 generated to bind the ASK1 in ΔNcGrx1 parasites. Eventually, ASK1 is continuously activated, and induction of apoptosis.

Our study found that NcGrx1 deficiency leads to slower intracellular growth owing to the weakened invasion and egress abilities. The previous study showed that the microneme proteins (MICs) play a key role in the invasion and egress of the apicomplexan parasite (Frénal et al., 2017). MIC4-MIC1-MIC6 exists in a complex (Reiss et al., 2001; Li et al., 2015). The absence of MIC4, MIC1, or MIC6 affects the function of the complex and the invasion of the parasite (Brecht et al., 2001; Cérède et al., 2005; Zheng et al., 2009). CDPK1 is found to play a critical role in calcium-regulated secretion in micronemes, resulting in a strong reduction in host cell invasion, and egress (Lourido et al., 2010). SUB1 is a serine protease that mediates the processing and adhesive properties of microneme proteins during invasion (Lagal et al., 2010). The AMA1-RON2 complex is demonstrated as an important components of the moving junction, which is essential for invasion process (Gaur and Chitnis, 2011; Lamarque et al., 2014). Our study found that the absence of NcMIC6 reduced the egress capacity of *N. caninum* (unpublished). NcGrx1 deletion resulted in the downregulation of transcriptional levels of invasion-related factors, and the secretion and processing of MIC1, MIC4, and MIC6 proteins. This causes substantial impairment in parasite growth. Interestingly, NcSUB1 transcriptional levels were also reduced in ΔNcGrx1 parasites, which suggested that NcGrx1 may regulate NcSUB1 at the transcriptional level. Therefore, this study suggests that NcGrx1 may regulate the secretion and processing of microneme proteins by affecting the expression of SUB1.

Dithiothreitol (DTT) is a small molecule organic reducing agent that reduces disulfide bonds. Since DTT is not present in nature, glutaredoxin and thioredoxin are reported as the most abundant cellular reducing dithiol catalyst (Stommel et al., 1997). Exposure of the tachyzoites to DTT triggers the release of calcium ions, which causes the parasites to egress. After the treatment of ΔNcGrx1 and Nc1 with DTT, we found that the transcript and secretion level of MIC1, MIC4, and MIC6 in ΔNcGrx1 recovered. This further confirmed that NcGrx1 contributed to the reduced secretion of microsomal proteins, which may be related to its redox function. Remarkably, the transcriptional level of the NTPase3 gene in Nc1 was significantly increased in the DTT-treated group as compared with ΔNcGrx1, while the untreated group was equivalent in both ΔNcGrx1 and Nc1. NTPases are enzymes with apyrase activity. NTPase activity may be regulated by an oxidoreduction change in its molecule caused by a dithiol compound or an unknown dithiol-disulfide oxidoreductase within the parasitophorous vacuole (Stommel et al., 1997; Asai and Tomavo, 2007). DTT can activate *N. caninum* NTPase, and significantly enhance the transcriptional level of NTPase (Asai et al., 1998; Pastor-Fernández et al., 2016). In this study, NcGrx1 deletion did not change the transcription level of NTPase when stimulated with DTT. This is consistent with the current assumptions about Grxs and the egress mechanism of *T. gondii*. It has been postulated that the NTPase might be activated by Grxs. The NTPases increasingly deplete host cell ATP by abating Na^+^/K^+^-ATPase pumps, decreasing K^+^, and triggering egress (Stommel et al., 2001; Asai and Tomavo, 2007; Blader et al., 2015; Pastor-Fernández et al., 2016).

In summary, we identified two *N. caninum* Grxs localized in the cytoplasm. The deletion of NcGrx1 parasites displayed a significant growth defect, which was due to the influence of invasion and egress ability. The loss of NcGrx1 resulted in the downregulation of multiple invasion-egress related factors. Further investigation found that the secretion of MIC1, MIC4, and MIC6 proteins was significantly decreased. These causes significant impairment in parasite growth *in vitro*. Moreover, the loss of NcGrx1 led to decline in the GSH/GSSG ratio, the accumulation of excessive hydroxyl radical and induction of apoptosis in parasites, which suggested that NcGrx1 is crucial to the maintenance of redox homeostasis in *N. caninum* under oxidative stress.

## Data Availability Statement

All datasets generated for this study are included in the article/Supplementary Material.

## Ethics Statement

The animal experiments were in strict accordance with the recommendations of the Guide for the Care and Use of Laboratory Animals of the Ministry of Science and Technology of China. All experimental procedures were approved by the Institutional Animal Care and Use Committee of China Agricultural University (under the certificate of Beijing Laboratory Animal employee ID: 18049).

## Author Contributions

QL, XS, and JL conceived and designed the study. XS performed the experiments. QL and XS analyzed the data and drafted the manuscript. XY and JL helped in manuscript writing. YX helped in bioinformatics analysis. CY helped in plasmid construction. KW helped in animal experiments. All authors read and approved the final manuscript.

## Conflict of Interest

The authors declare that the research was conducted in the absence of any commercial or financial relationships that could be construed as a potential conflict of interest.

## Footnotes

1 https://toxodb.org/toxo/

2 http://expasy.org/

3 http://smart.embl-heidelberg.de/

4 http://swissmodel.expasy.org

5 https://pymol.org/2/

6 http://vina.scripps.edu/

## Supplementary Material

FIGURE S1 PCR identification of iΔNcgrx1 parasites. **(A)** Strategy for construction of NcGrx1 complementary strain (iΔNcGrx1). **(B)** PCR identification of iΔNcGrx1. **(C)** Plaque assay comparing the growth of wild-type, knockout, and complementary parasites.

FIGURE S2 The activity of recombinant NcGrx1 at different concentrations were assayed as the decrease in absorption at 340 nm at 25 °C (ΔAbs/min).

TABLE S1 Database information of *Neospora caninum* glutaredoxins.

TABLE S2 Primers used for this study.

TABLE S3 The primers of invasion-egress related genes used for qPCR.

TABLE S4 Database accession numbers.
