# Supplementary figures and images for: Glutaredoxin 1 Deficiency Leads to Microneme Protein-Mediated Growth Defects in Neospora caninum
Source: Front Microbiol. 2020 Aug 31;11:536044. doi: 10.3389/fmicb.2020.536044 (PMC7487798; doi:10.3389/fmicb.2020.536044)

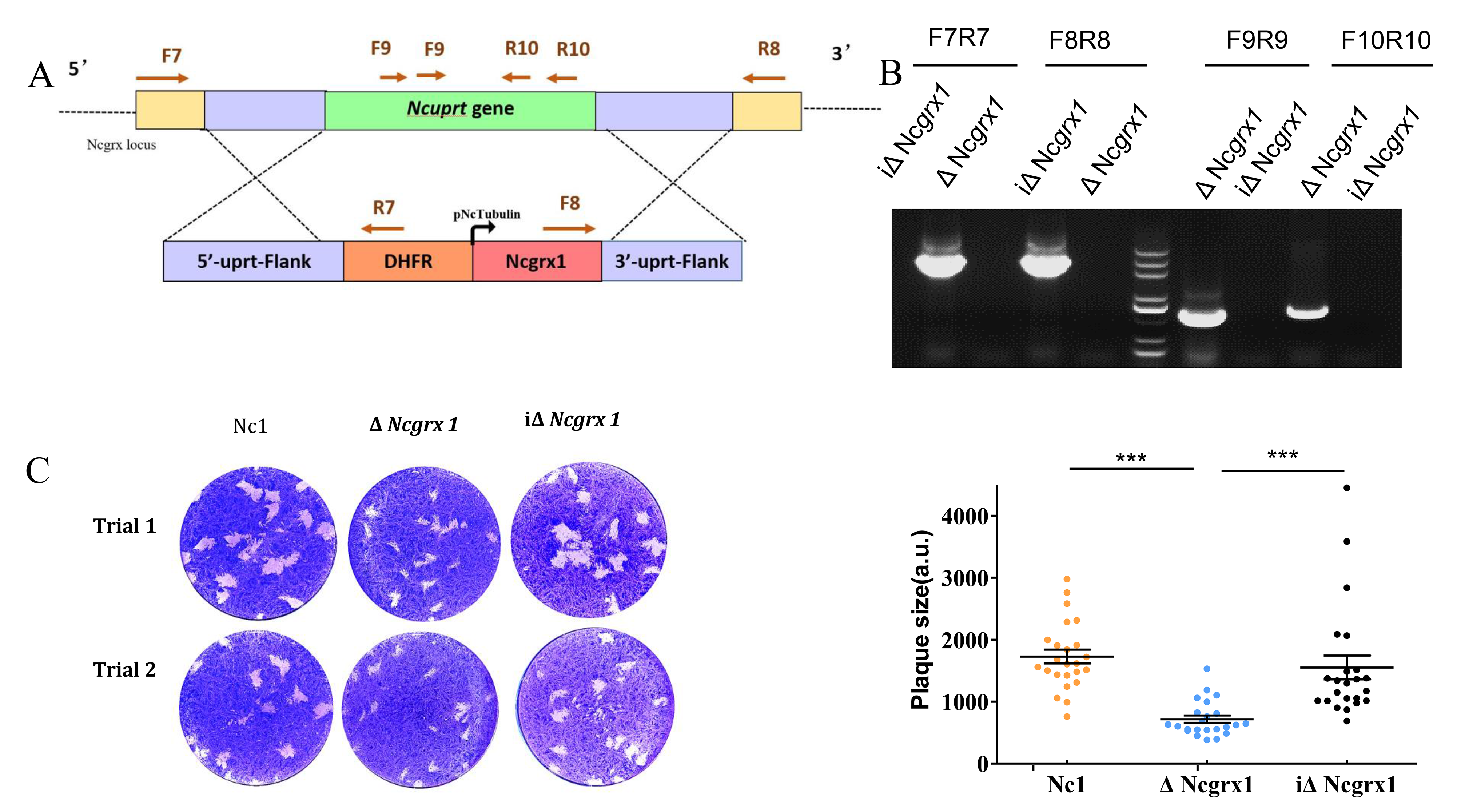

Supplement: FIGURE S1 — PCR identification of iΔNcgrx1 parasites. (A) Strategy for construction of NcGrx1 complementary strain (iΔNcGrx1). (B) PCR identification of iΔNcGrx1. (C) Plaque assay comparing the growth of wild-type, knockout, and complementary parasites. [file Image_1.TIF]

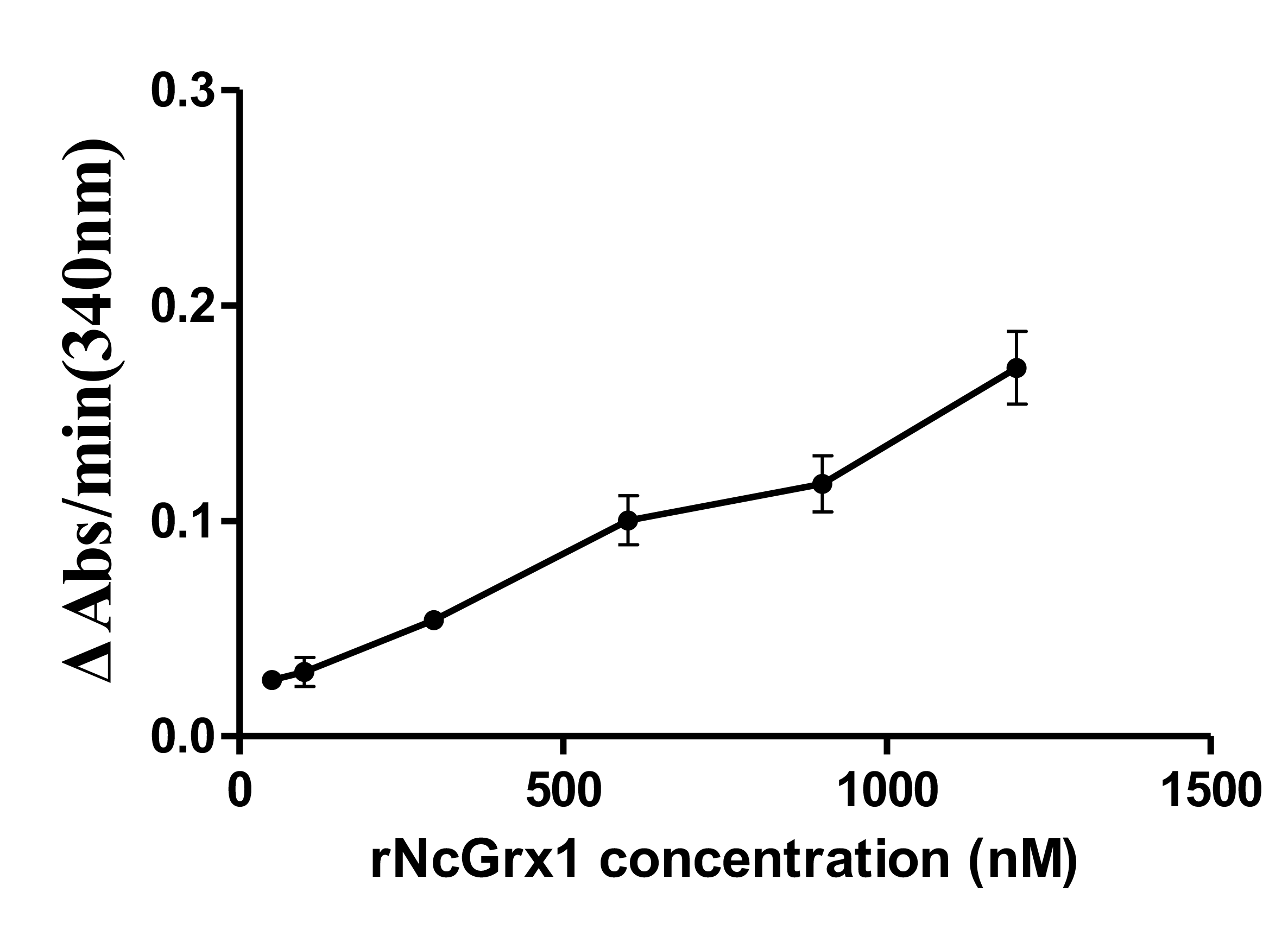

Supplement: FIGURE S2 — The activity of recombinant NcGrx1 at different concentrations were assayed as the decrease in absorption at 340 nm at 25 °C (ΔAbs/min). [file Image_2.TIF]
