# Supplementary material for: Glutaredoxin 1 Deficiency Leads to Microneme Protein-Mediated Growth Defects in Neospora caninum
Source: Front Microbiol. 2020 Aug 31;11:536044. doi: 10.3389/fmicb.2020.536044 (PMC7487798; doi:10.3389/fmicb.2020.536044)
Supplement: TABLE S1 — Database information of Neospora caninum glutaredoxins. [file Table_1.DOCX]

**Supplementary Table 1** **Database information of *Neospora caninum* glutaredoxins**

|  | Ncgrx1 | Ncgrx3 |
| --- | --- | --- |
| Gene ID | NCLIV_038390 | NCLIV_015460 |
| cDNA (bp) | 339 | 717 |
| Protein length | 112 | 238 |
| Molecular weight (kDa) | 12.4 | 26.4 |
| Isoelectric point | 6.78 | 5.35 |
| SignaIP | no | no |
