# Supplementary material for: Glutaredoxin 1 Deficiency Leads to Microneme Protein-Mediated Growth Defects in Neospora caninum
Source: Front Microbiol. 2020 Aug 31;11:536044. doi: 10.3389/fmicb.2020.536044 (PMC7487798; doi:10.3389/fmicb.2020.536044)
Supplement: TABLE S2 — Primers used for this study. [file Table_2.DOCX]

**Supplementary Table 2** **Primers used for this study**

| **Primer name** |  | **Sequence** |
| --- | --- | --- |
| Cas9-primer | F | 5′- ATACGACTCACTATAGGGCG -3′ |
|  | R | 5′- AGCTCCACCGCGGTGGCGGC -3′ |
| ΔNcGrx1-U6 | F | 5′- GCCGCCACCGCGGTGGAGCT -3′ |
|  | R | 5′- AAGATGGGGATCCTCGGAATAAACAACAATGTCCCTTTGG -3′ |
| ΔNcGrx1- gRNA-AMP | F | 5′- ATTCCGAGGATCCCCATCTTGTTTTAGAGCTAGAAATAGC -3′ |
|  | R | 5′- CGCCCTATAGTGAGTCGTAT -3′ |
| ΔNcGrx1- 3’flank | F | 5′- TCAAATATTGACGAAAGAAC -3′ |
|  | R | 5′- CACGCGAGAGACGGCAGCT -3′ |
| ΔNcGrx1- 5’flank | F | 5′- CATCACGGTCACAAGAGAAT -3′ |
|  | R | 5′- CTTTGAATCGTCCAAATAGA -3′ |
| ΔNcGrx1-RFP-CAT | F | 5′- TCTATTTGGACGATTCAAAGTATAGGGCGAATTGGGTACC-3′ |
|  | R | 5′- GTTCTTTCGTCAATATTTGAGATATCTCTAGTGGATCCCC -3′ |
| ΔNcGrx1-AMP | F | 5′- AGCTGCCGTCTCTCGCGTGCCTAGGGCTAGCTCTAGAACT-3′ |
|  | R | 5′- ATTCTCTTGTGACCGTGATGGCCGTCGTTTTACAACGTCG-3′ |
| ΔNcGrx3-U6 | F | 5′- GCCGCCACCGCGGTGGAGCT -3′ |
|  | R | 5′- TCCAAGGCTGGTATCCCTATAAACAACAATGTCCCTTTGG -3′ |
| ΔNcGrx3-gRNA-AMP | F | 5′-ATAGGGATACCAGCCTTGGAGTTTTAGAGCTAGAAATAGC -3′ |
|  | R | 5′- CGCCCTATAGTGAGTCGTAT -3′ |
| ΔNcGrx3- 3’flank | F | 5′-AACAACAGCACGGAAAGCCC -3′ |
|  | R | 5′- AGAGTTACGCACCTTCTCTG-3′ |
| ΔNcGrx3- 5’flank | F | 5′-TGTCTGTGTCTGTCGAGGCA-3′ |
|  | R | 5′- ATTTCCCCTTCTTCGGCACC-3′ |
| ΔNcGrx3-RFP-CAT | F | 5′- ATTTCCCCTTCTTCGGCACCCTAGCATGTCATTCGATTTT-3′ |
|  | R | 5′- GGGCTTTCCGTGCTGTTGTTACTAGTGGATCGATCCCCCG -3′ |
| ΔNcGrx3-AMP | F | 5′- CAGAGAAGGTGCGTAACTCTCCTAGGGCTAGCTCTAGAAC-3′ |
|  | R | 5′- TGCCTCGACAGACACAGACAGCCGTCGTTTTACAACGTCG-3′ |
| NcGrx1(HA)-U6 | F | 5′- GCCGCCACCGCGGTGGAGCT -3′ |
|  | R | 5′- CGTACAGTCTCTGATCCACGcAAACAACAATGTCCCTTTGG-3′ |
| NcGrx1(HA)- gRNA-  AMP | F | 5′-gCGTGGATCAGAGACTGTACGGTTTTAGAGCTAGAAATAGC-3′ |
|  | R | 5′- CGCCCTATAGTGAGTCGTAT -3′ |
| NcGrx1(HA)- 3’flank | F | 5′-CGGGGGATCGATCCACTAGTCTGCTTTTGAGAGATTCGT-3′ |
|  | R | 5′- GCGGTGGCGGCCGCTCTAGATCCCAACTACCCGCCCTCTC-3′ |
| NcGrx1(HA)- 5’flank | F | 5′-TCCAATTTAATTAAGATATCTGATGCCCGTGTGTGCTAGG-3′ |
|  | R | 5′- ACGTCGTACGGGTACCTAGGCAGAGCTCCAGCTGCTCGAA-3′ |
| NcGrx1(HA)-DHFR | F | 5′- CCTAGGTACCCGTACGACGT-3′ |
|  | R | 5′- ACTAGTGGATCGATCCCCCG-3′ |
| NcGrx1(HA)-AMP | F | 5′- TCTAGAGCGGCCGCCACCGC-3′ |
|  | R | 5′- GATATCTTAATTAAattgga-3′ |
| NcGrx3 (HA)-U6 | F | 5′- GCCGCCACCGCGGTGGAGCT -3′ |
|  | R | 5′- CCAAAGGGACATTGTTGTTTGCATTTACGCCCATTGCACAG-3′ |
| NcGrx3(HA)-gRNA-AMP | F | 5′- GCATTTACGCCCATTGCACAGGTTTTAGAGCTAGAAATAGC-3′ |
|  | R | 5′- CGCCCTATAGTGAGTCGTAT -3′ |
| NcGrx3 (HA)- 3’flank | F | 5′- GTAGCGAGACAGTCGCGTTC-3′ |
|  | R | 5′- AGAGTTACGCACCTTCTCTG-3′ |
| NcGrx3 (HA)- 5’flank | F | 5′- GTTTGAGAGCCCACCTTCAA-3′ |
|  | R | 5′- GGCGAAGGCCTCCGGCGGGAACA-3′ |
| NcGrx3(HA)-DHFR | F | 5′- TGTTCCCGCCGGAGGCCTTCGCCCCTAGGTACCCGTACGACGT-3′ |
|  | R | 5′- GAACGCGACTGTCTCGCTACACTAGTGGATCGATCCCCCG-3′ |
| NcGrx3(HA)-AMP | F | 5′- CAGAGAAGGTGCGTAACTCTTCTAGAGCGGCCGCCACCGC-3′ |
|  | R | 5′- TTGAAGGTGGGCTCTCAAACGATATCTTAATTAAATTGGATTGG-3′ |
| NcUPRT-U6 | F | 5′- GCCGCCACCGCGGTGGAGCT -3′ |
|  | R | 5′- CCAAAGGGACATTGTTGTTTGCGGGCGAGTCGATGGAAAG-3′ |
| NcUPRT-gRNA-AMP | F | 5′- GCGGGCGAGTCGATGGAAAGGTTTTAGAGCTAGAAATAGC-3′ |
|  | R | 5′- CGCCCTATAGTGAGTCGTAT -3′ |
| NcGrx1(OE)-5’-UPRT-flank | F | 5′- AAACGACGGCCAGTGAATTCCTCTGACGGGGCTCTTCCTA-3′ |
|  | R | 5′- CTTGTCAACGTCATCGCCATTTTGTCGAAAAAGGGAATTC-3′ |
| NcGrx1(OE)-3’-UPRT-flank | F | 5′- TTCGAGCAGCTGGAGCTCTGTACCCGTACGACGTCCCGGA-3′ |
|  | R | 5′- TAGGAAGAGCCCCGTCAGAGGAATTCACTGGCCGTCGTTT-3′ |
| NcGrx1(OE) | F | 5′- ATGGCGATGACGTTGACAAGC-3′ |
|  | R | 5′- CAGAGCTCCAGCTGCTCGAA-3′ |
| NcGrx3(OE)-5’-UPRT-flank | F | 5′- AAACGACGGCCAGTGAATTCCTCTGACGGGGCTCTTCCTA-3′ |
|  | R | 5′- GTTTGCATGCTGCTCGCCATTTTGTCGAAAAAGGGAATTC-3′ |
| NcGrx3(OE)-3’-UPRT-flank | F | 5′- TCCCGCCGGAGGCCTTCGCCTACCCGTACGACGTCCCGGA-3′ |
|  | R | 5′- TAGGAAGAGCCCCGTCAGAGGAATTCACTGGCCGTCGTTT-3′ |
| NcGrx3(OE) | F | 5′- ATGGCGAGCAGCATGCAAAC-3′ |
|  | R | 5′- GGCGAAGGCCTCCGGCGGGA-3′ |
| NcGrx2 | F1 | 5′- AGCTTGTCGCTAGCTTCCAC-3′ |
|  | R1 | 5′- ACTGCGAACAGCAGCAAGAT-3′ |
|  | F2 | 5′- CAGATGTGCGTGTATCCACT-3′ |
|  | R2 | 5′- GGTACGAAGCGGAGAGAAAT-3′ |
|  | F3 | 5′- ATGAGCAGTGAAACGCTAGG-3′ |
|  | R3 | 5′- GCAACTGACTGAAATGCCTC-3′ |
|  | F4 | 5′- ATGTCGGGCGAAACCTCGAA-3′ |
|  | R4 | 5′- GGCGGTTAATGAAGACATGC-3′ |
|  | F5 | 5′- ACTCTGCTCCGTCGTCA-3′ |
|  | R5 | 5′- CGTTTCCATCGCTCACTT-3′ |
|  | F6 | 5′- ATGAAGCAGGCGAGAAA-3′ |
|  | R6 | 5′- AGCCGAATACGATGAACT-3′ |
| NcGrx3 | F1 | 5′- GTAGAGTCGACACAGTTCGA-3′ |
|  | R1 | 5′- ACTGCGAACAGCAGCAAGAT-3′ |
|  | F2 | 5′- CAGATGTGCGTGTATCCACT-3′ |
|  | R2 | 5′- TATCTACGCCTCCTTGGCGC-3′ |
|  | F3 | 5′- ATGTCGGGCGAAACCTCGAA-3′ |
|  | R3 | 5′- GCAACTGACTGAAATGCCTC-3′ |
|  | F4 | 5′- ATGTCGGGCGAAACCTCGAA-3′ |
|  | R4 | 5′- CGATTCAGGTTCATCATGCC-3′ |
|  | F5 | 5′- ATGCTTTGCTCACGCCTGCA-3′ |
|  | R5 | 5′- AAGCTCTGACTGCCTGGACG-3′ |
|  | F6 | 5′- TCTGAGCAACGGAGCGAGTT-3′ |
|  | R6 | 5′- CTGGCAAGTCTCTCCTGGAG-3′ |
| iΔNcGrx1-5’flank | F7 | 5′-CCCCACGCTCTATCACGCGAG-3′ |
|  | R7 | 5′- TTGCGGTGTCGTGGATTTAC-3′ |
| iΔNcGrx1- 3’flank | F8 | 5′- GCAGACAACTTTCCTTCTATGCAC-3′ |
|  | R8 | 5′- GATAGTTGTTCTGCTAACTCGTGGG-3′ |
| iΔNcGrx1- uprt1 | F9 | 5′- GACTGCGCAGGATATATACT-3′ |
|  | R9 | 5′- TCGTTTCCGTACCTTGGGCT-3′ |
| iΔNcGrx1- uprt2 | F10 | 5′- CGCTCGTGACTTTCTGTTTT-3′ |
|  | R10 | 5′- AAAGGAGAGTGGACAGCGAG-3′ |
