# Supplementary material for: Glutaredoxin 1 Deficiency Leads to Microneme Protein-Mediated Growth Defects in Neospora caninum
Source: Front Microbiol. 2020 Aug 31;11:536044. doi: 10.3389/fmicb.2020.536044 (PMC7487798; doi:10.3389/fmicb.2020.536044)
Supplement: TABLE S3 — The primers of invasion-egress related genes used for qPCR. [file Table_3.DOCX]

**Supplementary Table 3: The primers of invasion-egress related genes used for qPCR.**

| NcMIC1-F | GCGGAGTTGATAGGGATAA |
| --- | --- |
| NcMIC1-R | GGGCAGGACCAGGTTGTT |
| NcMIC2-F | GACTTGTTCACTGCCTCCAT |
| NcMIC2-R | TTCGTCTCCTGCGTTCTT |
| NcMIC3-F | GAACCACTGAAGGCAAGAAC |
| NcMIC3-R | GGGAAGTCATAGAACGGGAG |
| NcMIC4-F | GCTGCCTAATGCTGCTGA |
| NcMIC4-R | ATTCGCTGGCTGCGGACT |
| NcM2AP-F | AACCTCGCTGCCGTGTC |
| NcM2AP-R | CCCATCGTGCCCTGAAC |
| NcMIC6-F | GAGACAATACAGACCGAACAC |
| NcMIC6-R | CCCAACAGCAAGAGGC |
| NcMIC8-F | CATTGTAGCTGCAACCCA |
| NcMIC8-R | CATCAGGCAACACTCTTTT |
| NcCDPK1-F | GAAAGTTGAGAAAGGCAAATAC |
| NcCDPK1-R | CGTTGTCAAGGGAGGGTA |
| NcAMA1-F | TCCACTGACATCGCAACA |
| NcAMA1-R | GCCTTAGAATCCGAAACC |
| NcSUB1-F | GAGGAGCATCGTGGTTTC |
| NcSUB1-R | CCCGCAGGCTTGTCTA |
| NcRON2-F | CGGGACAAGTCAAGTTCTACCA |
| NcRON2-R | TTCGCTTCCTGAGCCATC |
| NcNTPase3-F | AACCGTCATCCATTTCCT |
| NcNTPase3-R | ATCCCGTCTCATAAGCTGTA |
| NcActin-F | TGTACATCGAAGGAAATCCC |
| NcActin -R | GAACCGATTCCCAGCATCCA |
