# Supplementary material for: Glutaredoxin 1 Deficiency Leads to Microneme Protein-Mediated Growth Defects in Neospora caninum
Source: Front Microbiol. 2020 Aug 31;11:536044. doi: 10.3389/fmicb.2020.536044 (PMC7487798; doi:10.3389/fmicb.2020.536044)
Supplement: TABLE S4 — Database accession numbers. [file Table_4.DOCX]

**Supplementary Table 4. Database accession numbers**

*Neospora caninum* glutaredoxin 1 (NcGrx1; ToxoDB: NCLIV_038390), *Mus musculus* glutaredoxin 1 (MmGrx1; GenBank: NP_444338.2), *Homo sapiens* glutaredoxin 1 (HsGrx1; GenBank: AAC35798.1), *Caenorhabditis elegans* glutaredoxin (CeGrx1; GenBank: NP_490812.1), *Plasmodium falciparum* glutaredoxin 1 (PfGrx1; GenBank: BAB79691.1), *Toxoplasma gondii* glutaredoxin (TgGrx1; ToxoDB: TGGT1_279400), *Arabidopsis thaliana* glutaredoxin (AtGrx1; GenBank: NP_198853.1), and *Cryptosporidium parvum* glutaredoxin (CpGrx1; GenBank: XP_626456.1).
